# PGC-1α Is Required for Exercise- and Exercise Training-Induced UCP1 Up-Regulation in Mouse White Adipose Tissue

**DOI:** 10.1371/journal.pone.0064123

**Published:** 2013-05-22

**Authors:** Stine Ringholm, Jakob Grunnet Knudsen, Lotte Leick, Anders Lundgaard, Maja Munk Nielsen, Henriette Pilegaard

**Affiliations:** Department of Biology, Centre of Inflammation and Metabolism and August Krogh Centre, University of Copenhagen, Copenhagen, Denmark; National Institutes of Health, United States of America

## Abstract

**Background:**

The aim of the present study was to test the hypotheses that 1) a single exercise bout increases UCP1 mRNA in both inguinal (i)WAT and epididymal (e)WAT, 2) UCP1 expression and responsiveness to exercise are different in iWAT and eWAT, 3) PGC-1α determines the basal levels of UCP1 and PRDM16 in WAT and 4) exercise and exercise training regulate UCP1 and PRDM16 expression in WAT in a PGC-1α-dependent manner.

**Methods:**

Whole body PGC-1α knockout (KO) and wildtype (WT) littermate mice performed a single treadmill exercise bout at 14 m/min and 10% slope for 1 hour. Mice were sacrificed and iWAT, eWAT and quadriceps muscle were removed immediately after, 2, 6 and 10 hours after running, and from sedentary mice that served as controls. In addition, PGC-1α KO mice and WT littermates were exercise trained for 5 weeks with sedentary mice as untrained controls. Thirty-six-37 hours after the last exercise bout iWAT was removed.

**Results:**

UCP1 mRNA content increased 19-fold in iWAT and 7.5-fold in eWAT peaking at 6 h and 0′ of recovery, respectively, in WT but with no changes in PGC-1α KO mice. UCP1 protein was undetectable in eWAT and very low in iWAT of untrained mice but increased with exercise training to 4.4 (AU) in iWAT from WT mice without significant effects in PGC-1α KO mice.

**Conclusion:**

The present observations provide evidence that exercise training increases UCP1 protein in iWAT through PGC-1α, likely as a cumulative effect of transient increases in UCP1 expression after each exercise bout. Moreover, the results suggest that iWAT is more responsive than eWAT in exercise-induced regulation of UCP1. In addition, as PRDM16 mRNA content decreased in recovery from acute exercise, the present findings suggest that acute exercise elicits regulation of several brown adipose tissue genes in mouse WAT.

## Introduction

Life style related metabolic diseases are an increasing problem worldwide and is often associated with obesity and adipose tissue malfunction. Adipose tissue is an endocrine organ playing an important role in whole body metabolism. Several studies [1], [2] indicate that inguinal white adipose tissue (iWAT), opposite of epididymal white adipose tissue (eWAT), has a protective effect on metabolic diseases. Therefore, the amount and distribution of adipose tissue seem important in development of metabolic diseases. Furthermore, inguinal- and epididymal-derived cell lines have been reported to exhibit different responsiveness to Forskolin/cAMP stimulation [3] and iWAT has recently been shown to contain beige precursor adipocytes [3], [4] while eWAT does not. This indicates potential different responses in eWAT and iWAT to certain stimuli.

Stallknecht et al. [5], [6] showed that WAT is able to adapt to endurance exercise training much like skeletal muscle. Hence Cytochrome c oxidase (COX) activity [5] and GLUT4 mRNA content [6] increased in rat eWAT after 10 weeks of endurance exercise training. In addition, an acute exercise bout has been shown to induce gene responses in adipose tissue from mice and rats [7], [8] suggesting that cumulative effects of transient increases in mRNA lead to these adaptations. Furthermore, the uncoupling protein (UCP)1 mRNA content has been shown to increase in iWAT but not in eWAT in mice in response to cold-exposure [9] and in both adipose tissue depots with exercise training but most markedly in iWAT [10]. No changes were evident in iWAT UCP1 mRNA in response to acute exercise in mice in that study [10]. However, only one time point was measured (5 h of recovery), and UCP1 mRNA was not measured in eWAT after a single exercise bout. In addition, exercise training-induced changes in UCP1 protein in WAT remains to be proven. Protein-containing PR (PRD1-BF-1-RIZ1 homologous) domain (PRDM)16 has also been identified as a regulator of the brown fat-like gene program and thermogenesis in iWAT [11], [12], but whether this protein is regulated in white adipose tissue in response to exercise is currently unresolved.

It is at present unknown which factors are regulating the exercise-induced UCP1 response in WAT. The transcriptional co-activator peroxisome proliferator-activated receptor-γ coactivator (PGC)-1α has previously been shown to drive the formation of brown fat gene program [13] in addition to playing a role in regulation of capillarization [14], [15] and expression of oxidative proteins in skeletal muscle [15], [16] and oxidative proteins in adipocytes [17]. Muscle PGC-1α has recently been suggested to influence UCP1 expression through PGC-1α mediated regulation of irisin release from skeletal muscle [10]. However, whether PGC-1α is required for exercise-mediated regulation of UCP1 expression in WAT is unresolved. Therefore, the aim of the present study was to test the hypotheses that 1) a single exercise bout induces UCP1 mRNA responses in both iWAT and eWAT, 2) PGC-1α determines the basal levels of UCP1 and PRDM16 in iWAT and eWAT, 3) exercise up-regulates UCP1 and PRDM16 mRNA in iWAT and eWAT in a PGC-1α-dependent manner and 4) exercise training up-regulates UCP1 protein in iWAT in a PGC-1α-dependent manner.

## Methods

### Mice

The study used whole body PGC-1α knockout (KO) and wildtype (WT) littermate mice. PGC-1α KO and WT mice were obtained by intercross breeding of heterozygous parents [18] and homozygous offspring were used for experiments. During the experimental period, the mice were housed individually in cages with 12∶12-h light-dark cycle and with free access to standard chow (Altromin, Brogården ApS, Lynge, Denmark) and water.

### Experimental Protocol

#### Acute exercise bout

Prior to the experimental day, mice were acclimatized to treadmill exercise (TSE systems GmbH, Bad Homburg, Germany) two times 10 min a day on five consecutive days. Each 10 min exercise period consisted of 2 min at 8 m/min, 2 min at 10 m/min, 4 min at 15 m/min and 2 min at 10 m/min, with a constant slope of 10%.

Forty-eight hours after the end of adaptation to treadmill running, PGC-1α KO mice and WT littermates performed a single 1 hour treadmill exercise bout at 14 m/min with 10% slope and both genotypes completed the exercise bout, although PGC-1α KO mice exercised at a relatively higher intensity [19]. Mice were sacrificed by cervical dislocation immediately after (0 h), 2 (2 h), 6 (6 h) or 10 (10 h) hours after running, while mice not run acutely served as controls (Rest). Inguinal (iWAT), which are found anterior to the upper segment of the hind limb, and epididymal (eWAT), found underneath the abdomen skin, white adipose tissue and quadriceps muscle were quickly removed and frozen in liquid nitrogen for later analyses.

#### Exercise training

In addition to the acute exercise bout, a group of PGC-1α whole body KO and WT littermates were exercise trained for 1 hour 5 times/week for 5 weeks and had access to running wheels during the exercise period as previously described [15], [19] with a control group not training. Running wheels were blocked occasionally in WT mice to ensure similar total running duration per day in WT and PGC-1α KO mice as previously published [19]. Mice were sacrificed by cervical dislocation 36–37 h after the last exercise bout and iWAT, was removed and quickly frozen in liquid nitrogen.

### Analyses

#### Muscle glycogen

Muscle glycogen content was determined from 15 mg of muscle tissue as glycosyl units after acid hydrolysis [20] using a fluoroscan (Thermo Labsystems, Bie & Berntsen, Rødovre, Denmark).

#### RNA isolation and reverse transcription

Total RNA was isolated from ∼30 mg of adipose tissue by a modified guanidinium thiocyanate-phenol-chloroform extraction method adapted from Chomczynski and Sacchi [21] as previously described [22] except that the tissue was homogenized for 2 min at 30 sec^–1^ in a TissueLyserII (Qiagen, Valencia, CA, USA).

Superscript II RNase H^–^ system and Oligo dT (Invitrogen, Carlsbad, CA, USA) were used to reverse transcribe the mRNA to cDNA as previously described [22].

#### Real-time PCR

The mRNA content of UCP1 and PRDM16 were determined by real time PCR using the fluorogenic 5′ nuclease assay with TaqMan probes and universal mastermix with UNG (ABI PRISM 7900 Sequence Detection System, Applied Biosystems, CA, USA) as previously described [23]. The sequences used to amplify a fragment of UCP1 were FP: 5′AAGCGTACCAAGCTGTGCGA3′, RP: 5′AGAAAAGAAGCCACAAACCCTT3′ and TaqMan probe: 5′CCATGTACACCAAGGAAGGACCGACG3′ and to amplify a fragment of PRDM16 were FP: 5′CAGCACGGTGAAGCCATTC3′, RP: 5′GGCGTGCATCCGCTTGT3′ and TaqMan probe: 5′ATGCGAGGTCTGCCACAAGTCCTACAC3′. Both TaqMan probes were 5′-6-carboxyfluorescein (FAM) and 3′-6-carboxy-N,N,N’,N’-tetramethylrhodamine (TAMRA) labeled. The obtained cycle threshold (Ct) values reflecting the initial content of the specific transcript in the samples were converted to an arbitrary amount by using standard curves obtained from a serial dilution of a pooled sample made from all samples. The amount of a given mRNA was normalized to the ssDNA content of the cDNA sample determined by use of OliGreen as previously described [23].

#### Lysate preparation

Adipose tissue specimens were homogenized in an ice-cold buffer as previously described [24] except the tissue was homogenized for 2 min at 30 sec^–1^ in TissueLyserII (Qiagen, Valencia, CA, USA). Protein content in lysates was measured by the bicinchoninic acid method (Pierce Biotechnology Inc., Rockford, IL, USA). Lysates were prepared with sample buffer containing Sodium dodecyl sulfate (SDS) and boiled for 3 min at 96°C and analyzed by SDS-PAGE and western blotting.

#### SDS-PAGE and western blotting

Protein content was measured in adipose tissue samples by SDS-PAGE and western blotting using PVDF membrane and semi-dry transfer as previously described [24]. Protein content is expressed in units relative to control samples loaded on each gel. Primary UCP1 (ab10983 Abcam), COXIV (ab16056 Abcam) and CD31 (SC-1506 Santa Cruz) antibodies and polyclonal secondary antibodies (Dako, Glostrup, Denmark) were used.

#### Statistics and calculations

Values presented are means ± SE. Two-way analysis of variance was applied to test the effect of acute exercise and genotype on mRNA, protein content and muscle glycogen as well as the effect of exercise training and genotype on UCP1 protein content using the Student-Newman-Keuls post hoc test to locate differences. Differences were considered significant at P≤0.05. Statistical calculations were performed using SigmaPlot version 11.0.

## Results

### Acute Exercise

#### Muscle glycogen content

Resting muscle glycogen content was similar in WT and PGC-1α KO and muscle glycogen was reduced (P≤0.05) 25 and 65% after the acute exercise bout in WT and PGC-1α KO, respectively, with no significant difference between genotypes (Table 1).

**Table 1:** Muscle glycogen content at rest (Rest) and immediately after exercise (0 h).

	WT	PGC-1α KO
**Rest**	20.75±2.46	19.46±1.70
**0 h**	15.59±1.32*	6.53±1.01*

Skeletal muscle glycogen content at rest (Rest) and immediately after (0 h) an acute exercise bout from whole body PGC-1α knockout (KO) and wildtype (WT) littermate mice. Values are means±SE; n = 8.

* Significantly different from Rest within given genotype, P≤0.05.

#### UCP1 mRNA content

**Figure 1:**
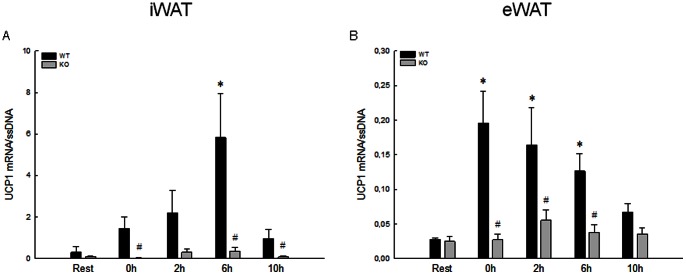
UCP1 mRNA content in iWAT and eWAT in response to acute exercise. Uncoupling protein (UCP) 1 mRNA content in iWAT (A) and eWAT (B) immediately after (0 h), 2 (2 h), 6 (6 h) and 10 (10 h) hours after an acute exercise bout and from rested (Rest) wildtype (WT) and whole body PGC-1α knockout (KO) mice. UCP1 mRNA is normalized to single stranded (ss) DNA. Values are means±SE; n = 8. *: Significantly different from Rest within given genotype, P≤0.05. ^#^: Significantly different from WT within given time point, P≤0.05.

The resting content of UCP1 mRNA was in both iWAT and eWAT similar in WT and PGC-1α KO (Figure 1A and B). The basal Ct level was on average ∼31 and ∼37 in iWAT and eWAT, respectively.

In WT, the mRNA content of UCP1 increased (P≤0.05) ∼19-fold in iWAT (Figure 1A) at 6 hours of recovery relative to Rest and ∼7-fold in eWAT (Figure 1B) immediately after the acute exercise bout relative to Rest, but with no changes in PGC-1α KO mice (Figure 1A and 1B).

#### PRDM16 mRNA content

**Figure 2:**
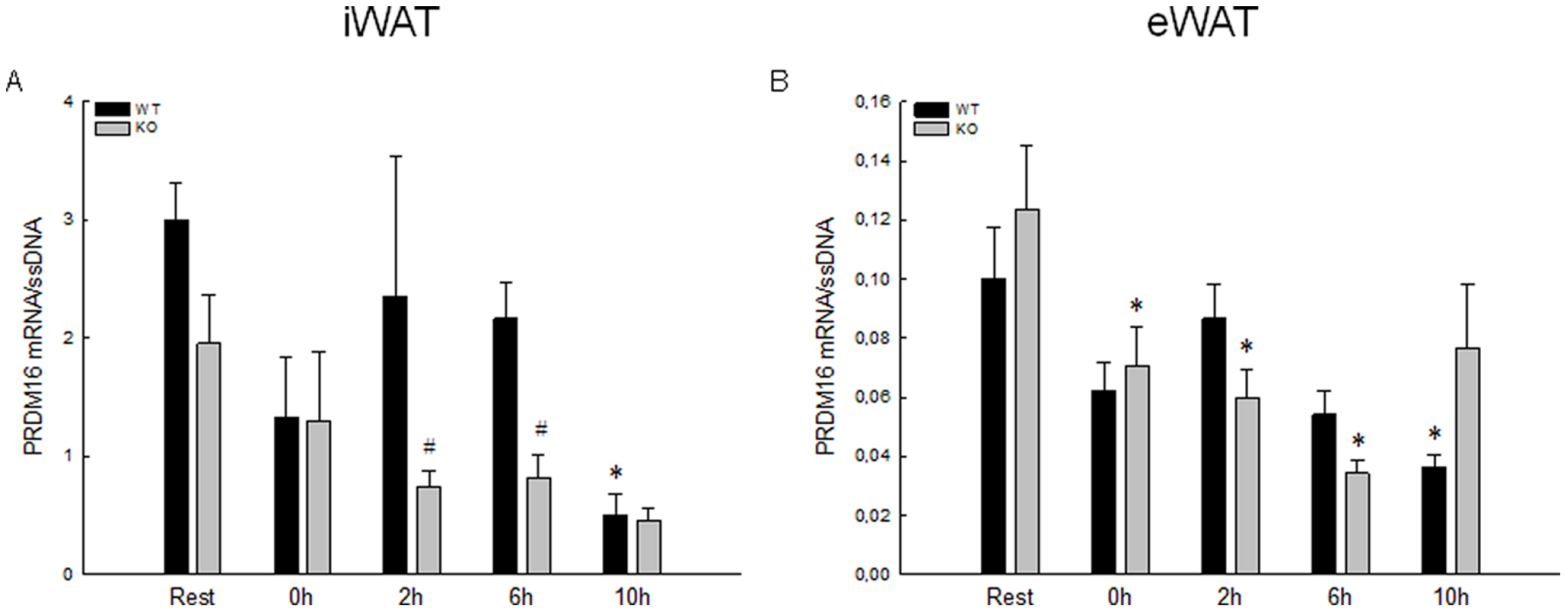
PRDM16 mRNA content in iWAT and eWAT in response to acute exercise. Protein-containing PR (PRD1-BF-1-RIZ1 homologous) domain (PRDM) 16 mRNA content in iWAT (A) and eWAT (B) immediately after (0 h), 2 (2 h), 6 (6 h) and 10 (10 h) hours after an acute exercise bout and from rested (Rest) wildtype (WT) and whole body knockout (KO) mice. PRDM16 mRNA is normalized to single stranded (ss) DNA. Values are means±SE; n = 8. *: Significantly different from Rest within given genotype, P≤0.05. ^#^: Significantly different from WT within given time point, P≤0.05.

The resting content of PRDM16 mRNA was in both iWAT and eWAT similar in WT and PGC-1α KO mice (Figure 2A and B).

In WT mice, the PRDM16 mRNA content decreased (P≤0.05) in both iWAT and eWAT at 10 h of recovery from the acute exercise bout to ∼20–30% of the level in Rest, while in PGC-1α KO mice the PRDM16 mRNA content decreased (P≤0.05) at 0 h, 2 h and 6 h of recovery from the acute exercise bout to ∼30–60% of the level in Rest, only in eWAT. In addition, the PRDM16 mRNA content in iWAT was at Rest, 2 h and 6 h of recovery 30–45% lower (P≤0.05) in PGC-1α KO than in WT mice (Figure 2A and 2B).

#### UCP1 protein content

**Figure 3:**
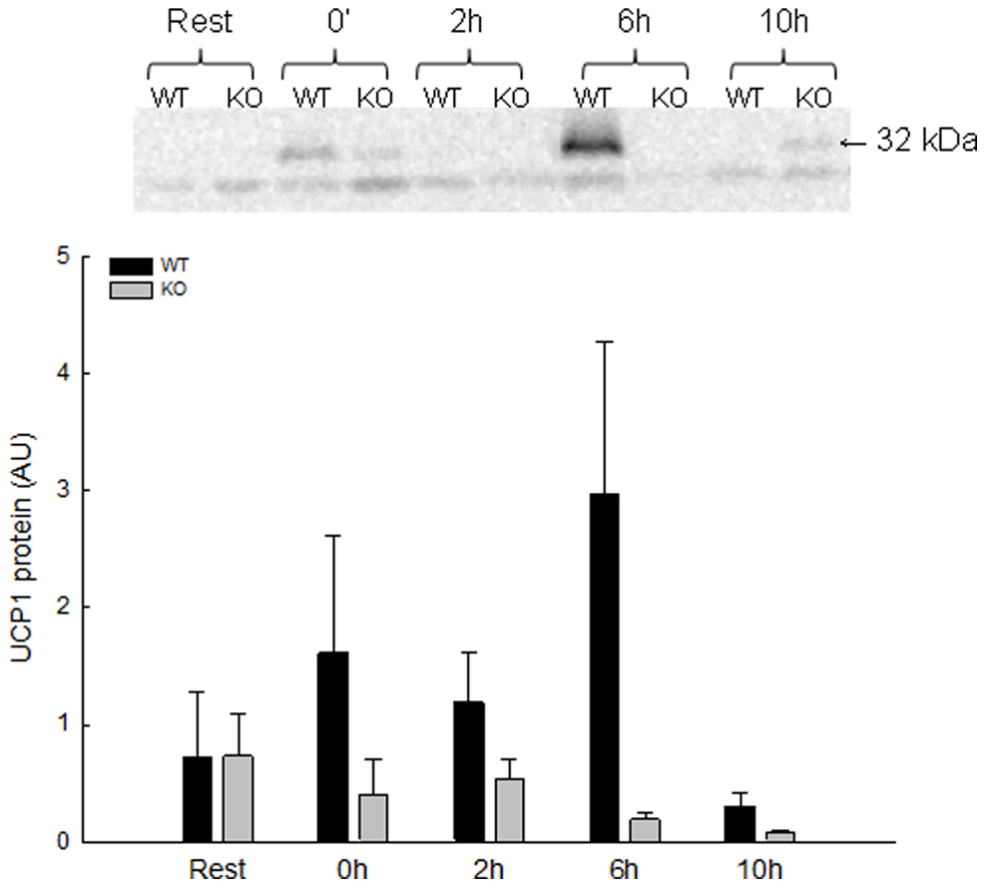
UCP1 protein content in iWAT in response to acute exercise. Uncoupling protein (UCP) 1 protein content in iWAT immediately after (0 h), 2 (2 h), 6 (6 h) and 10 (10 h) hours after an acute exercise bout and from rested (Rest) wildtype (WT) and whole body PGC-1α knockout (KO) mice given in arbitrary units (AU). Values are means±SE, n = 8.

The resting content of UCP1 protein in iWAT was undetectable in 25% of the resting samples from WT and PGC-1α KO mice taken together, while it was undetectable in all eWAT samples (Figure 3).

UCP1 protein content in iWAT did not change significantly during recovery from the acute exercise bout (Figure 3).

### Exercise Training

#### UCP1 protein content

**Figure 4:**
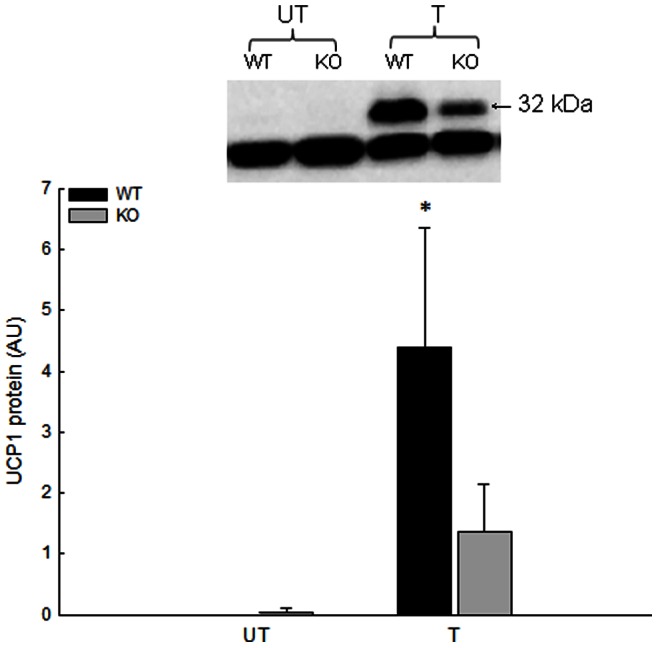
UCP1 protein content in iWAT in response to exercise training. Uncoupling protein (UCP) 1 protein content in iWAT from untrained (UT) and trained (T) wildtype (WT) and whole body PGC-1α knockout (KO) mice given in arbitrary units (AU). Values are means±SE, n = 8. *: Significantly different from UT within given genotype, P≤0.05.

The protein content of UCP1 in iWAT was undetectable in approximately 75% of the untrained samples in WT and PGC-1α KO mice taken together and increased (P≤0.05) to 4.4 (A.U.) in trained WT, but did not change in PGC-1α KO (Figure 4).

#### COXIV protein content

The protein content of COXIV in iWAT was ∼3-fold higher (P≤0.05) in untrained PGC-1α KO than in untrained WT mice. COXIV protein content in iWAT was ∼2.5-fold higher (P≤0.05) in trained WT than in untrained WT mice, while there was no change in PGC-1α KO with exercise training (Table 2).

**Table 2:** COXIV and CD31 protein content in iWAT in response to exercise training.

	Untrained		Trained	
	WT	PGC-1α KO	WT	PGC-1α KO
**COXIV**	0.3±0.1	0.8±0.2#	0.7±0.1*	0.9±0.1
**CD31**	0.4±0.1	0.9±0.1#	0.7±0.1	1.0±0.2

COXIV and CD31 protein content (arbitrary units) in iWAT from untrained and trained whole body PGC-1α knockout (KO) and wildtype (WT) mice. Values are means±SE; n = 8.

* Significantly different from untrained within given genotype, P≤0.05.

# Significantly different from WT within given time point, P≤0.05.

#### CD31 protein content

The protein content of CD31 in iWAT was ∼2-fold higher (P≤0.05) in untrained PGC-1α KO than in untrained WT mice. CD31 protein content in iWAT was non-significantly ∼1.6-fold higher (P = 0.079) in trained WT than in untrained WT mice, while there was no change in PGC-1α KO with exercise training (Table 2).

## Discussion

The findings of the present study demonstrate transient exercise-induced UCP1 mRNA responses in mouse iWAT and eWAT, but with different time course of the response. Furthermore, UCP1 protein content increased with exercise training in iWAT. In addition, PGC-1α was required for both acute and exercise training-induced regulation of UCP1 in WAT.

The present study shows for the first time that exercise elicited a transient UCP1 mRNA increase in both iWAT and eWAT of WT mice in recovery from an acute exercise bout and the study demonstrates the time course of exercise-induced UCP1 mRNA responses in iWAT and eWAT. Furthermore, the novel observations that iWAT UCP1 protein content was higher in trained than in untrained WT mice in the present study add to the recent findings that the UCP1 mRNA content in iWAT and eWAT increased with exercise training in mice [10] and suggest that exercise training-induced UCP1 expression in iWAT may have functional significance. The demonstrated transient increase in UCP1 mRNA content in iWAT from WT mice makes it possible that the observed long term protein adaptations are accumulations from the repeated transient gene responses.

The lack of exercise-induced increases in UCP1 mRNA in both eWAT and iWAT of whole body PGC-1α KO mice indicates that PGC-1α is required for the acute exercise-induced regulation of UCP1 mRNA in WAT. The observed similar reduction in muscle glycogen content in WT and PGC-1α KO mice in response to the acute exercise bout supports that the PGC-1α KO mice have been physically challenged as the WT and that the lack of UCP1 response therefore is not due to lack of exercise stimulus. In addition, the long term UCP1 protein adaptations in iWAT with exercise training seem to require PGC-1α. However, the observation that the resting level of UCP1 protein in iWAT did not differ between WT and PGC-1α KO mice indicates that PGC-1α is not needed for the basal UCP1 levels in WAT, although the very low basal UCP1 protein level makes this comparison difficult. A PGC-1α independent basal UCP1 level may also seem in contrast to the previous observation that muscle-specific PGC-1α overexpression mice had elevated iWAT UCP1 mRNA content [10]. However, as previous studies [16], [19], [25] have suggested that PGC-1α is involved but not necessarily required for exercise training-induced adaptations in mitochondrial proteins in skeletal muscle, muscle-specific PGC-1α overexpression mice may be seen as a model of exercise trained animals. The results may therefore indicate that basal UCP1 expression is independent of PGC-1α, while exercise-induced UCP1 regulation requires PGC-1α.

The present findings, that exercise training also increased the content of the oxidative protein, COXIV, and the capillarization marker, CD31, in iWAT in WT but not in PGC-1α KO mice, further suggest that PGC-1α exerts a concerted regulation of capillarization, oxidative proteins and UCP1 expression in iWAT with exercise training in mice. However, the higher basal COXIV and CD31 protein levels in iWAT of PGC-1α KO mice than WT is different from previous suggestions of PGC-1α mediated up-regulation of oxidative capacity in adipose tissue [7], [26]. This may suggest that a compensatory mechanism is in play in iWAT of the PGC-1α KO mice leading to increased oxidative capacity of iWAT without clear effects on basal UCP1 expression. In addition, the different observations in Kleiner et al. [26], in adipose tissue-specific PGC-1α KO mice, and the present study in whole body PGC-1α KO mice, may be due to the different mouse models.

The current observations of PCR cycle threshold (Ct) levels for basal UCP1 mRNA around ∼31 for iWAT and ∼37 for eWAT, demonstrate that the UCP1 mRNA level is markedly higher in iWAT than in eWAT with a hardly detectable level in eWAT. In addition, the present notion that UCP1 protein is undetectable in eWAT and in most samples also in iWAT is in accordance with a recent study by Wu et al. [3] showing that in the basal state UCP1 protein is only detectable in brown adipose tissue and not in iWAT and eWAT. In addition, the observed differences in fold change of UCP1 mRNA to acute exercise with 19-fold in iWAT and 7-fold in eWAT is in accordance with a recent study [10] showing that, after exercise training the relative mRNA content in iWAT is 22-fold higher than in eWAT, suggesting different responsiveness of the two adipose tissue depots to acute exercise. In addition, the present study identifies the time course of the exercise-induced UCP1 mRNA response in iWAT peaking at 6 h while the UCP1 mRNA content in eWAT was peaking immediately after the acute exercise bout. The different time courses may contribute to different abilities for long term adaptations, because the longer lasting response in iWAT increases the chance for mRNA accumulation with repeated bouts of exercise [27].

The present finding that the PRDM16 mRNA content decreased in recovery from the acute exercise bout while UCP1 mRNA increased suggests that acute exercise elicited a response similar to cold-exposure with increased UCP1 and decreased PRDM16 expression [9]. In addition, the present observations does not suggest a role of PGC-1α in the regulation of PRDM16 mRNA content in recovery from the acute exercise bout, while PGC-1α seems at least in part involved in determining the basal PRDM16 mRNA content in iWAT.

In conclusion, the present results demonstrating that UCP1 mRNA in both iWAT and eWAT increases in response to a single exercise bout and that exercise training increased UCP1 protein in iWAT add to previous reports and support that exercise induces an up-regulation of UCP1 expression in WAT. The findings that basal UCP1 mRNA and/or protein in iWAT and eWAT was similar in PGC-1α KO and WT mice indicate that PGC-1α is not required for basal UCP1 expression in WAT. However, the increase in UCP1 mRNA in iWAT and eWAT with acute exercise and UCP1 protein in iWAT of WT, but not PGC-1α KO provides evidence that PGC-1α is mandatory for exercise mediated regulation of UCP1 expression in iWAT. The functional role of such changes is unknown and additional studies are required to address this.
